# Impacts of Poultry House Environment on Poultry Litter Bacterial Community Composition

**DOI:** 10.1371/journal.pone.0024785

**Published:** 2011-09-16

**Authors:** Michael D. Dumas, Shawn W. Polson, Don Ritter, Jacques Ravel, Jack Gelb, Robin Morgan, K. Eric Wommack

**Affiliations:** 1 Department of Biological Sciences, University of Delaware, Newark, Delaware, United States of America; 2 Department of Plant and Soil Sciences, University of Delaware, Newark, Delaware, United States of America; 3 Delaware Biotechnology Institute, University of Delaware, Newark, Delaware, United States of America; 4 Mountaire Farms Inc., Millsboro, Delaware, United States of America; 5 Institute for Genome Sciences, University of Maryland School of Medicine, Baltimore, Maryland, United States of America; 6 Department of Animal and Food Sciences, University of Delaware, Newark, Delaware, United States of America; 7 Center for Bioinformatics and Computational Biology, University of Delaware, Newark, Delaware, United States of America; Argonne National Laboratory, United States of America

## Abstract

Viral and bacterial pathogens are a significant economic concern to the US broiler industry and the ecological epicenter for poultry pathogens is the mixture of bedding material, chicken excrement and feathers that comprises the litter of a poultry house. This study used high-throughput sequencing to assess the richness and diversity of poultry litter bacterial communities, and to look for connections between these communities and the environmental characteristics of a poultry house including its history of gangrenous dermatitis (GD). Cluster analysis of 16S rRNA gene sequences revealed differences in the distribution of bacterial phylotypes between Wet and Dry litter samples and between houses. Wet litter contained greater diversity with 90% of total bacterial abundance occurring within the top 214 OTU clusters. In contrast, only 50 clusters accounted for 90% of Dry litter bacterial abundance. The sixth largest OTU cluster across all samples classified as an *Arcobacter* sp., an emerging human pathogen, occurring in only the Wet litter samples of a house with a modern evaporative cooling system. Ironically, the primary pathogenic clostridial and staphylococcal species associated with GD were not found in any house; however, there were thirteen 16S rRNA gene phylotypes of mostly Gram-positive phyla that were unique to GD-affected houses and primarily occurred in Wet litter samples. Overall, the poultry house environment appeared to substantially impact the composition of litter bacterial communities and may play a key role in the emergence of food-borne pathogens.

## Introduction

Advances in technology over the last century have greatly increased the scale of both crop and livestock agriculture. In the past 50 years, poultry production and consumption of broiler meat has increased by approximately 4% per year [1]. In the United States, poultry consumption in 2009 was nearly 21 billion kg of meat produced from 8.2 billion broiler chickens valued at 21 billion US dollars [2]. Steady production increases have resulted from industry moves to ever-larger poultry houses with most containing more than 20,000 birds.

Despite its economic benefit, the high stocking density of birds in a house has spawned numerous health issues for both birds and humans [3]. In part, these issues arise from the volume of litter produced in a poultry house. Plant-based bedding material along with chicken excrement, feathers, and spilled feed are the principal components of litter. Typically, in United States broiler houses, a layer of new bedding material is deposited between each new flock and over the course of several years; dozens of flocks will be raised on a single bed of layered litter. Thus, poultry litter likely maintains the microbiological record of every past flock and is believed to be a reservoir of disease-causing microorganisms.

Previous molecular genetic studies detected a diverse range of antibiotic resistance genes [4] and both human and avian pathogens [5] within poultry litter samples. However, none of these studies has investigated possible links between the composition of litter microbial communities and the incidence of disease within a poultry house. One emerging disease of concern in the poultry industry is gangrenous dermatitis (GD), an avian disease that demonstrates links in its occurrence to environmental factors [6]. This low morbidity, high mortality disease progresses rapidly and begins with redness or swollen areas on the skin which quickly progress to large gangrenous lesions of dead and dying tissue [7]. Once the first symptoms occur, infected individuals die within 24–72 hours. The primary pathogens associated this disease are *Clostridium perfringens*, *Clostridium septicum*, and *Staphylococcus aureus* [8], [9], [10],. However, there is no consensus on how the disease is spread or why some poultry houses exhibit chronic recurring outbreaks while other houses nearby never experience an outbreak. Moreover, in the US, GD incidence is most prevalent during the late spring/early summer in the Delmarva growing region of the mid-Atlantic, with other geographic regions experiencing little to no incidence of the disease [6]. Because affected and unaffected houses all receive the same bedding material, feed and medication regimen, and broiler chicken breed, a variable that may contribute to GD incidence is the composition of litter microbial communities within a house.

The recent use of high-throughput sequencing methods has enabled the study of bacterial communities with an unprecedented amount of depth and clarity. Deep sequencing studies have consistently found bacterial taxa not detected through traditional cultivation-based analyses in both environmental [11], [12], [13], [14], [15], and clinical samples [16], [17], [18]. These studies have begun to uncover a much greater richness of microbial taxa within target environments and enabled better definition of the compositional structure of a microbial community. Through these data we now appreciate that the sum of less abundant taxa can collectively make up a significant fraction of the total microbial population and play a role in regulating the overall heath of an organism or ecosystem.

To obtain a more comprehensive picture of poultry litter bacterial communities and to examine potential connections between these communities, the environment within a poultry house, and the incidence of recurring GD outbreaks, we employed 454 pyrosequencing of 16S rRNA gene PCR amplification products derived from poultry litter. Analyses of poultry litter bacterial 16S rRNA gene sequences included a combination of both *a priori* and *a posteriori* bioinformatic approaches such that all sequences were included regardless of homology to previously characterized sequences. These data were placed in the larger context of poultry litter microbial ecology through the inclusion of both bacterial and viral abundance data using direct counting methods.

## Materials and Methods

### Ethics statement

Proprietors of each poultry house provided permission and access for the collection of litter samples.

### Sample collection

Samples were collected on a single day in August, 2008 from four poultry houses of four different farms in the Delmarva Peninsula. All four houses were under contract by the same company and received bedding material and feed from the same distributor. A 15 cm spade rinsed with 70% ethanol was used to collect 4 scoops of litter from the top 3–6 inches of litter within in a 5-meter area and placed in 1 gal zip-loc bags. The process was repeated in another part of the house between 5 and 25 meters away and placed in a separate bag. Litter directly under the water lines was collected in the same manner as the Dry samples and roughly parallel to where they were collected. All 16 samples were stored at 4°C until DNA extraction and enumeration.

### Determining Moisture Content

Samples were weighed using an analytical balance (Mettler Toledo) and dried in a vacuum oven (Lindberg Blue) at approximately 100 degrees Celsius for 48 h. Dry weight was subtracted from the initial weight to determine percent moisture content.

### DNA Extraction

All samples were homogenized by hand and divided into two sub-samples of approximately equal weight. The duplicate samples from each house and litter type were pooled (to increase the in-house coverage) giving a total of 4 Dry and 4 Wet litter samples. Sterile PBS was used to bring the Dry litter to the same consistency as the Wet litter so that approximately equal masses of each sample would be subjected to extraction. An enzyme cocktail optimized to lyse Gram-positive bacteria was mixed with the litter samples [consisting of 0.15 g of litter, 5 µl lysozyme (10 mg/ml), 15 µl Mutanolysin (11.7 U/µl), 33 µl lysostaphin (4.24 U/µl), 10 µL proteinase K (20 mg/ml), 50 µL 10% SDS in 1 mL of 0.05 potassium phosphate buffer]. The mixture was shaken in a FastPrep FP120 (MP Bio) instrument for 40 s and allowed to sit for 5 min. DNA from the mixture was then purified using Zymo-Spin IV-HRC spin filters and accompanying kit reagents. DNA concentration of the elutant was measured by a nanodrop ND-1000 spectrophotometer (Thermo Scientific), aliquoted, and stored at −20° C.

### Sequencing

Each of the 8 samples was amplified using a barcoded universal bacterial 16S rRNA gene reverse primer with adaptors for 454 pyrosequencing (Roche) (Table S1) as described by [19]. Following bacterial genomic DNA extraction, the V1–V2 hypervariable region of the bacterial small-subunit ribosomal RNA gene was PCR amplified from each sample. All samples used the same forward primer with accompanying 454 linkers. The components for one, 25 µL PCR reaction are as follows: 0.1 µL Platinum Taq High Fidelity (Invitrogen), 2.5 µL 10× high fidelity PCR buffer, 1 µL 50 mM MgSO4, 0.5 µL 10 mM dNTP Mix, 0.75 µL forward primer, 5 µL reverse primer, 50 ng amplified DNA sample (not to exceed 10 uL), 5.15 µL nuclease-free water. PCR conditions are as follows: 94° C for 2 min, 94° C for 30 s, 52° C for 30 s, 68° C for 1 min (repeat temperature 2–4 30 times), 68° C for 5 min. Amplifiication products and negative controls were run on 2 separate 1% agarose gels (made with TAE and ethidium bromide) for 35 min at 105 V. A BioRad Geldoc XR system and accompanying software was used to determine the DNA concentration of each amplification product. After the concentration was determined, 100 ng of DNA for each sample was pooled. Pooled amplification products for each house and sample type (8 total) were sequenced using a GS-FLX instrument (Roche).

### Bacterial Cell Extraction for Direct counts

In triplicate for each sample, 4 g of litter was weighed and placed in a 50 mL centrifuge tube. Following the procedure in van Elsas and Smalla [20] 40 ml of autoclaved 1% potassium citrate buffer (containing 10 g potassium citrate, 1.44 g Na_2_HPO_4_, 0.24 g KH_2_PO_4_, in 1 L H_2_0, pH: 7.0) was added to each tube, shaken for 5 s, and placed on ice for 10 min. The mixture was blended in a kitchen blender (Osterizer) for 3 min and transferred back to the centrifuge tube. Nine milliliters of the blended supernatant were transferred to an ultra centrifuge tube containing 2 mL Nycodenz (a density gradient media; Axis-Shield, Oslo, Norway) solution. The tubes were centrifuged at 10,000×g at 4° C using a SW 41 Ti rotor (Beckman Coulter). Supernatant (8.5 mL) was homogenized and transferred to two 4.5 mL cryovials, adjusted to 1% gluteraldehyde and snap frozen in liquid nitrogen.

### Viral Extraction for Direct Counts

Viruses were extracted according to [21] in triplicate by placing 5 g of litter in a 50 mL centrifuge tube followed by the addition of 15 mL of 1% potassium citrate buffer (containing 10 g potassium citrate, 1.44 g Na_2_HPO_4_, 0.24 g KH_2_PO_4_, in 1 L H_2_0, pH: 7.0). Tubes were vortexed for 5 s and placed on ice for 20 min. On ice, samples were sonicated at 100 W, 47 kHz (Branson S-450a) in three, 1 minute cycles, with 1 minute intervals in between each cycle. The mixture was then centrifuged at 3,000×g for 30 min. Supernatant from each sample was passed through a 0.22 µm sterivex filter (Millipore) into two to three 4.5 mL cryovials (depending on the amount of supernatant recovered) and snap frozen in liquid nitrogen.

### Bacterial/Viral Enumeration

One hundred microliter aliquots of virus or bacterial extract were diluted 1,000 to 10,000-fold in sterile deionized water and vacuum filtered (∼25 mm Hg) through a stack of 25-mm filters consisting of a 0.02-µm Anodisc filter (Whatman) for virus or 0.2 µm isopore membrane filter for bacteria (Millipore), a 0.22 µm Supor filter (Pall corporation), and a glass fiber filter (Pall Corporation). The anodisc or isopore filters were stained in the dark for 15 min with 400 µL of 1× SYBR Gold (Molecular probes). Filters were mounted on glass slides (Fisher Superfrost) along with 20 µL antifade solution (containing 20 mL PBS, 20 mL 100% glycerol, 400 µL p-phenyldiamine) to preserve fluorescent activity. Epifluorescent microscopy (EFM) was used to image the slides using an Olympus BX61 microscope (Olympus) with a flourescein isothiocynate excitation filter. Ten to fifteen fields per sample were imaged digitally at 1000× with a Retiga EXi camera (Q Imaging). Viruses were counted using iVision v4.0.8 software with a custom size-selection script. Bacteria were counted manually. Bacteria and virus counts for each sample type were averaged based on counts from three replicate slides.

### Sequence analysis

Raw 454 pyrosequences were separated and trimmed using the sample-specific barcode sequences described by Ravel et al. [19]. The quality of each sequence read was evaluated as described by Hamady et al. [22]. Each of the eight libraries were aligned using the NAST alignment tool available online from the Greengenes website (greengenes.lbl.gov). The minimum length was set at 200 bp and minimum identity at 75%.

ARB software v5.1 was used to generate a distance matrix for each library and for all libraries combined using the Jukes-Cantor substitution model. Using the ARB-generated distance matrices, DOTUR [23] was used to generate OTUs at 95% sequence identity for all libraries and for each library with rarefaction. Output files containing OTU frequency, Shannon-index and rarefaction curves were parsed using custom Perl scripts and used to generate figures and tables.

The Ribosomal Database Project (RDP) naïve Bayesian Classifier tool [24] was used to classify all sequences from the phylum through genus levels. The classifier was also used on the representative sequence of the most abundant and the unique OTUs generated from DOTUR. The RDP SeqMatch tool was used to compare representative sequences from individual OTUs to the RDP database. The BLASTn tool from NCBI was used to compare representative sequences from the top OTU clusters. To check whether chimeric 16S sequences made a significant contribution to the OTU clusters, representative sequences were analyzed using UChime [25] with a minimum score cut-off of 1.5 in *de novo* mode. This score is on the conservative end of the 0.1 to 5 minimum score recommended in the UChime documentation. In all, 96 clusters contained a putative chimeric representative sequence, the majority of which were singleton OTU clusters. These clusters accounted for 144 total sequences out of the 22,673 sequences collected.

Double principle coordinate analysis (DPCoA) [26] was performed on the sequences using R ver2.6.2 [27] with attached package ade4 [28].

## Results and Discussion

To date, no study has analyzed bacterial communities of poultry litter using deep sequencing of bacterial 16S rRNA gene amplification products. Previous studies investigating both litter and chicken intestinal microbial communities have employed denaturing gradient gel electrophoresis (DGGE), Sanger sequencing of 16S rRNA gene clone libraries, and cultivation-based assays (e.g., plate counting) [29], [30], [31], [32], [33]. With the depth of sampling that pyrosequencing allows; this research has resulted in the description of a litter microbial community with approximately 60-fold more sequence coverage than previous cultivation-independent 16S sequence studies [5].

Litter samples were collected from one house on each of four commercial poultry farms on the Delmarva peninsula. The farms were contracted with one broiler production company which supplied a standard corn, soybean based feed from a single commercial feed mill. Day to day husbandry practices were similar on all farms. The litter in each house was at least one year old and had been used to grow 5 to 6 consecutive flocks of chickens prior to sampling (Ritter pers. communication). Houses 1 and 2 had a history of recurring GD outbreaks, and were 30 or more years in age with suspended box fan ventilation (Table 1). Houses 3 and 4 had no history of GD and were younger, 10 and 20 years, respectively. House 3 had suspended box fan ventilation, whereas, the ventilation system in house 4 was changed to a more modern tunnel ventilation system with evaporative cooling (Table 1). Two samples types were collected from each house; 1) dry litter in the middle of the house, and 2) wet litter from underneath the water-dispensing lines. These two sample types are hereafter referred to as ‘Dry’ and ‘Wet’.

**Table 1:** General properties of each sample.

Sample name	Moisture content (%)	Bacterial abundance (g dry wt^−1^×10^10^) (SE)	Viral abundance (g dry wt^−1^×10^10^) (SE)	Virus to Bacteria ratio	Age of House in years	GD History	Ventilationsystem
Dry 1	25	2.4 (0.5)	5.0 (1.1)	2.1	30+	Yes	Suspended box fan
Wet 1	65	5.6 (0.5)	55.8 (14.6)	10.0			
Dry 2	22	2.5 (0.3)	25.5 (16.5)	10.2	30+	Yes	Suspended box fan
Wet 2	63	9.2 (1.4)	74.4 (17.4)	8.1			
Dry 3	19	1.3 (0.3)	ND^a^	ND	10+	No	Suspended box fan
Wet 3	67	4.6 (1.9)	199.4 (61.1)	43.4			
Dry 4	10	1.7 (0.4)	4.8 (1.5)	2.8	20^b^	No	Tunnel ventilation
Wet 4	43	4.6 (1.9)	94.2 (27.4)	20.5			

a No Data.

b Ventilation system changed 10 years ago.

### Microbiological and physical properties of poultry litter

Epifluorescence microscopy indicated that all litter samples contained around 10^10^ cells g dry wt^−1^ (Table 1). Mean bacterial abundance in wet litter samples was approximately three times higher than abundance in dry litter samples when normalized to cells per gram dry weight. Previous studies employing culture-based methods have provided inconsistent measurements of bacterial abundance with estimates ranging from 10^3^ to 10^12^ cells g^−1^ of litter, making comparison to direct counts difficult [29], [30], [31], [32], [33]. The only other litter study to use a culture independent method (qPCR quantitation of extracted bacterial DNA) estimated total bacterial abundances of 10^8^ to 10^10^ cells g^−1^ [34] levels comparable to the bacterial abundance results obtained by this study.

To date, no study has examined viral abundance in poultry litter by direct counting, although there have been numerous reports on the abundance of specific poultry and human viruses in litter and chicken [35], [36]. Across all litter samples viral abundance was 2 to 40-fold higher than corresponding bacterial abundance with values ranging from 10^10^ to 10^12^ viruses g dry wt^−1^ (Table 1). Similar to the trend observed in bacterial counts, viral counts and the virus to bacteria ratio was highest in wet litter samples (Table 1). These viral abundance values are between two and three logs greater than those found in various Delaware soils and Antarctic soil [37], [38] and five to six logs higher than lake and costal water [39]. Viral extracts from Dry litter samples also contained a higher proportion of what was assumed to be humic acids, which may have been responsible for our inability to obtain viral abundance data from the dry litter of house 3. Issues with the interference of humic acids have been reported previously in studies enumerating virus in both soils and sediments [21], [40], [41]. Despite this difficulty, this study has shown that viruses within poultry litter can be extracted and enumerated in a reproducible manner, thus paving the way for future cultivation-independent studies examining these viral communities.

With the exception of the litter underneath nipple drinkers, the moisture content in poultry litter should be fairly low and homogeneous throughout the house. Moisture content of dry litter samples ranged from 10–25% and wet litter from 43–67% (Table 1). Dry and wet litter moisture content in this study was similar to previous studies [30], [32], [42]. In addition, because litter underneath nipple drinker was saturated, a microaerophillic to anoxic microenvironment formed underneath the surface of wet litter [42]. Litter in House 4 had the lowest overall moisture content for both Dry and Wet samples, features likely attributable to the high forced ventilation rate within this house.

### Direct taxonomic classification of 16S gene libraries

This study employed pyrosequencing [43] of 16S rRNA gene libraries to analyze the bacterial composition of poultry litter. After processing for read quality and length, the eight libraries produced 22,673 sequences with an average read length of 236 bp. Individual library sizes ranged from 2,115 to 3,758 sequences (Table 2). 16S amplification product sequences were taxonomically classified using the classify tool available on the Ribosomal Database Project (RDP) website [44], [45]. Recent evidence suggests that for short reads covering only one or two variable regions, a 50% confidence cutoff maximizes the number of classifiable sequences in a library while maintaining high assignment accuracy [46]. Using these criteria, greater than 95% of the sequences were classified at the phylum through order levels, 85% at the family level, and 67% at the genus level (Table 3).

**Table 2:** Library clustering and unique clusters.

Sample name	Number of reads	Average read length^a^	Number of OTU clusters^b^	Unique^c^ OTU Clusters	Unique^d^ OTU clusters in both GD affected houses (total sequences)	Unique^d^ OTU clusters in both Non-GD affected Houses (total sequences)
Dry 1	2,616	240	156	122	13 (424)	
Wet 1	3,172	232	381			
Dry 2	2,115	238	197	88		
Wet 2	3,342	237	407			
Dry 3	2,499	238	202	96		12 (308)
Wet 3	2,849	233	325			
Dry 4	2,322	237	230	172		
Wet 4	3,758	236	529			

a Read length after trimming of primer and linker sequence.

b OTUs generated at 95% identity using UPGMA (average neighbor) clustering algorithm in DOTUR.

c Clusters found only in a single house after removal of all singleton clusters.

d Clusters at a frequency less than 0.05% were discarded in the target library and clusters less than 0.02% in comparison libraries were included if applicable.

**Table 3:** Diversity at different taxonomic levels.

Taxonomic level	Total no. all samples	Per library		% of library classified^a^ (SD)	% of total phylogeny^b^
		Range	Mean (SD)		
Phylum	9	4–7	5 (1)	99.2 (0.9)	23.7
Class	16	7–14	11 (3)	98.5 (1.6)	36.4
Order	38	8–29	18 (8)	97.0 (3.0)	39.2
Family	99	29–77	48 (17)	85.3 (5.3)	36.3
Genus	220	41–127	72 (32)	67.3 (10.4)	15.7

a Classified by RDP classifier at a bootstrap cutoff confidence interval of 50%.

b Based on the RDP classification scheme (total sequences phylogeny/total possible).

Because the composition of poultry litter bacterial communities has not been previously examined with this level of analytical depth, numerous taxa were observed that have never before been reported from poultry litter or the chicken intestine. The total number of RDP-classified taxa across all libraries was greater than the total taxa classified in any individual library, indicating the presence of sample-specific unique reads as high as the phylum level.

Previous cultivation and low throughput 16S rRNA studies reported the taxonomic composition of litter microbial communities within three broad classifications, high and low G-C Gram-positives and Gram-negatives [5], [29], [42], [47]. Although confounding factors such as flock size, litter age, and bedding material make comparisons difficult, previous studies indicated that poultry litter tends to have a high amount of Gram-positives with low G-C phyla dominating [5], [42], [47]. In this study, 77% of the RDP-classified sequences were assigned to Gram-positive taxa split into 44% low G-C and 33% high G-C phyla. This study found a greater frequency of Gram-negative phyla than previous reports. These differences were likely due to the sampling of wet litter that contained nearly all of the Gram-negative sequences. Although Lovnah *et al.* [42] noted a specific Dry-Wet split in the DGGE banding patterns of 16S amplification products from litter samples, subsequent sequence analysis did not indicate the presence of Gram-negative bacteria.

### House to house comparisons

**Figure 1:**
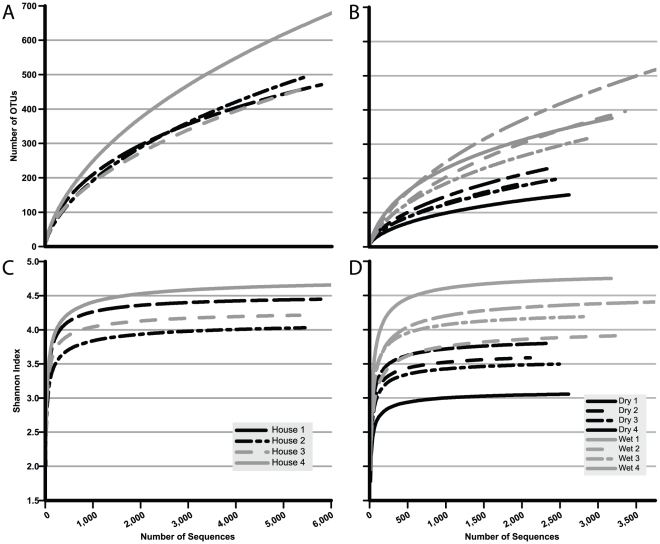
Rarefaction (A & B) and Shannon diversity index curves (C & D) for bacterial 16S rRNA gene OTUs at 95% similarity. A & C by poultry house; B & D by sample type and house number.

A total of 7,401 16S rRNA gene sequences could not be classified to the genus level at ≥50% confidence by RDP classifier. Using an OTU-based approach allowed for inclusion of all reads in a single analysis. At 95% sequence identity, a total of 1,462 OTU clusters were generated from the V1–V2 16S rRNA gene sequences. Removal singleton OTUs dropped this total to 777 clusters. House-to-house comparisons of bacterial richness by OTU rarefaction indicated overall OTU richness was increasing at ∼5,000 to ∼6,000 sequences (Fig. 1A); however, Shannon diversity was essentially flat after 2,000–3,000 sequences (Fig. 1C). Thus, increased sequencing would have only revealed rarer 16S rRNA gene OTUs and would not contribute significantly to diversity estimations.

**Figure 2:**
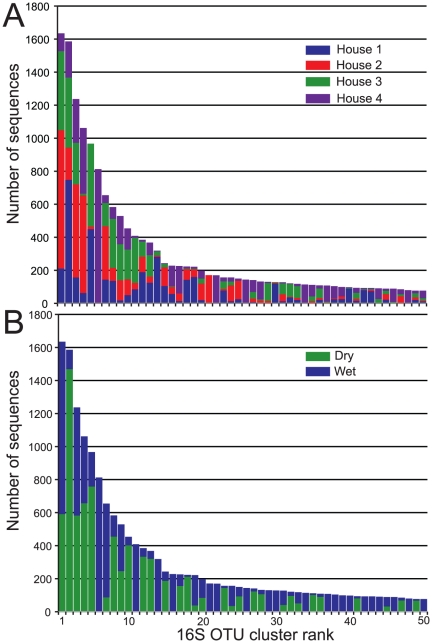
Rank abundance plots for the top 50 bacterial 16S rRNA gene OTUs split by house (A) and by sample type (B).

**Figure 3:**
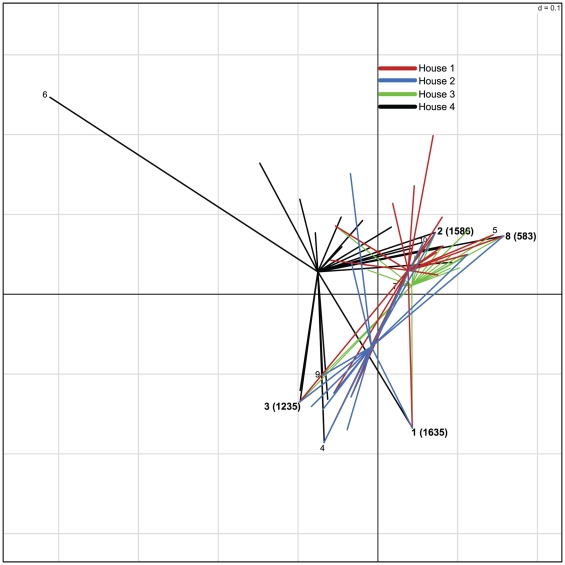
DPCoA displaying bacterial 16S rRNA gene OTUs with a frequency greater than 1% for each house. Positions of the ten most abundant OTUs are labeled and those shared by all houses are bold and accompanied by the total sequences in the OTU. The top two components covered 53.1 and 36.2 percent of the total variation on the X and Y axes respectively.

Rank abundance plots of 16S rRNA gene taxa or OTUs are often used to describe the structure of bacterial communities [48], [49]. OTU clusters were ranked by the number of sequences in the cluster to examine OTU distribution across all houses (Fig. 2A). Many of the top OTU clusters contained sequences from all houses, but the proportion of sequences from each individual house within a cluster differed. For example, in OTU cluster 1, roughly 50% of the sequences were from House 2, while cluster 2 was dominated by sequences from House 1. Other top OTU clusters were made up of sequences from only single house. Most notably, OTU clusters 6, 22 and 34, occurred only in House 4, and OTU cluster 21 in House 1. These house-to-house differences were apparent in double principle coordinate analysis (DPCoA) of the dataset [26]. DPCoA showed that houses diverged according to the identity and frequency of bacterial 16S rRNA gene OTUs within the litter, and that 90% of the variation between houses was explained by the top two components (Fig. 3).

Although each poultry house was given the same initial bedding material, feed, and antibiotic regimen, the conditions within each house were considerably different. The range of house conditions for broiler productions varies considerably between growers [3] and this study sought to sample a cross section of different houses on the Delmarva peninsula, one of the largest poultry growing regions in the U.S. (Table 1). Overall, these data indicate that house conditions have an impact on the composition of litter microbial communities. Houses 1 and 3 were dim with some ambient light from vents along the length of the house. The houses were kept cool using hanging fans and vents on the sides of the houses. These two houses shared roughly the same proportion of sequences in a number of the top 16S rRNA gene OTU clusters, including clusters 3, 5, 7, and 13 (Fig. 2A). House 2 was of similar age to House 1, but was more open, allowing an abundance of light to penetrate. Interestingly, a number of the most abundant OTUs contained a disproportionately large number of sequences from House 2 (e.g., clusters 1, 3, 4, 7, 21, 24, and 25). Perhaps greater exposure to environmental factors outside of this house had a beneficial influence on the already successful members of the microbial community. The substantially different environmental conditions resulting from the modern construction and evaporative cooling system likely influenced the divergence of the litter microbial communities in House 4 (Fig. 3).

The divergence of House 4 in DPCoA (Fig. 3) was of particular interest. This house had the highest richness and diversity according to 16S rRNA gene OTU analysis (Fig. 1A&C) and the largest number of unique OTU clusters (i.e., an OTU cluster containing of sequences from only one house) (Table 2). Furthermore, the proportions of the most abundant OTUs differed in House 4 as compared to the other three houses. There were 14 OTUs that comprised greater than 1% of the total sequences from House 4 and occurred at less than 1% abundance in any of the other houses (i.e. lines not converging with lines from another house) (Fig. 3). By comparison, the other houses had fewer of these “house specific” OTUs among the abundant OTUs (e.g., Houses 1 & 3 each had seven, House 2 had five among the top 1% clusters (Fig. 3)).

Examination of the 50 most abundant OTUs showed that the wet litter of House 4 contained unique OTUs not seen in any of the other houses (Fig. 2a & b). The 6^th^ largest 16S rRNA gene OTU was found only in the Wet litter of this house. This OTU classified to the genus *Arcobacter* in the family Campylobacteraceae [50]. *Arcobacter* is an emerging pathogen of concern in the poultry industry. This genus differs from *Campylobacter* in that these bacteria can tolerate oxygen and survive at lower temperatures. Like *Campylobacter*, *Arcobacter* is known to cause acute bacterial enteritis and improvements in medical diagnostics have revealed that *Arcobacter* infection can easily be misdiagnosed as *Campylobacter* infection [51], [52]. However, unlike *Campylobacter*, the route of transmission of *Arcobacter* contamination is poorly characterized. Studies focusing on the detection of arcobacterial contamination have obtained conflicting results on whether arcobacterial species are commonly found in the chicken gut [53], [54]. This is the first study to identify a large population of *Arcobacter* in poultry litter although some studies have identified it in broiler feces, a component of poultry litter [55]. Representative sequences of the top 50 OTU clusters and their RDP classification are given in the supplementary materials (Tables S2 and S3).

The 22^nd^ most abundant 16S rRNA gene OTU cluster, also found only in the Wet litter of House 4 (Fig. 2a), contained sequences most similar to the genus *Azospira*. These Gram-negative β-Proteobacteria are non-spore-forming with a polar flagellum . Currently, there are three described species of *Azospira* and a number of strains for *Azospira oryzae* [56], [57], [58]. This genus is of interest due to its potential for use in bioremediation. Strains of *Azospira* have been isolated that are able to reduce selenate and selenite to elemental selenium [59], and reduce the perchlorate to chloride [60]. Perchlorate reducing bacteria (putative *Azospira* sp and *Dechloromonas* sp) have been found in numerous soil and sediment environments [57] but this is the first study to report the presence of genus *Azospira* in a litter environment. The representative sequence for cluster 34 (unique to House 4) was classified by RDP to the genus *Dysgonomonas* (100% confidence), like *Azospria*, this is the first study to identify this genus in a litter environment.

These observations of highly abundant, but unique OTUs in House 4 raise the question of whether the more modern husbandry practice encourages growth of distinct litter microbial communities. These newer houses provide increased stability to the in-house environment and although this consistency is preferable for growing poultry, it may also promote a more virulent bacterial population [3]. Further sampling of a greater cross section of houses could shed light on their potential to host unique pathogens.

### Wet versus Dry litter

Few of the top 50 OTU clusters showed an even distribution of sequences between Dry and Wet litter samples, e.g., OTU clusters 3, 4, 9, 20, 25, and 33 (Fig. 2B). Twenty of the top 50 OTU clusters contained only Wet litter sequences, while none of the top 50 were composed of sequences from only Dry litter. Nevertheless, many bacterial phyla could survive in both microenvironments and indeed, the top five clusters all contained at least 100 sequences from both Dry and Wet libraries. Perhaps, the ability of these bacteria to survive in both conditions explains their dominance in the libraries.

**Figure 4:**
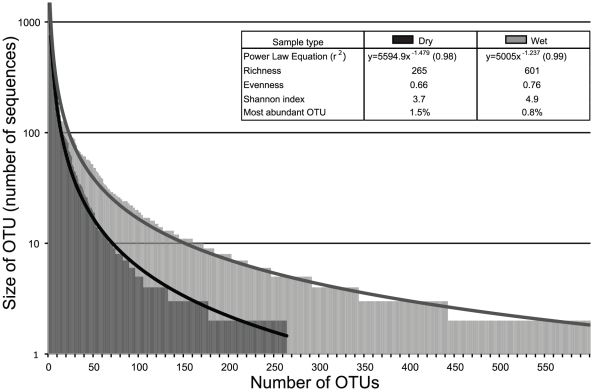
Bacterial 16S rRNA gene OTU rank abundance plots and power law curves fit for pooled Dry libraries (Black) and pooled Wet libraries (Gray). Singleton OTUs were removed prior to analysis. Richness (total OTUs observed), evenness (size distribution of OTUs), Shannon Diversity index, and most abundant OTU shown in table.

Rank abundance distribution curves of 16S rRNA gene OTUs within Dry and Wet samples showed that wet litter contained a higher richness and diversity of bacteria than dry litter (Fig. 4). While the curves follow a trend seen in communities of higher organisms (i.e. few, highly abundant organisms and many more rare organisms) [61], the inflection of the curve for each sample type was different (Fig. 4). According to the OTU rank abundance curves 90% of bacterial abundance in Dry litter occurred within the top 50 OTUs. In contrast, 214 clusters comprised the 90^th^ percentile of bacterial abundance in Wet litter. Compared proportionally, 90% abundance was covered by only 19% of the total Dry clusters, whereas 90% abundance in Wet litter was covered by 36% of the total clusters. Both the rarefaction and Shannon diversity index curves demonstrate that all the Wet libraries had greater richness and diversity than the Dry litter libraries (Fig. 1 B&D).

Most of the poultry litter 16S OTU clusters were small, containing one or two sequences. Other deep 16S pyrosequencing studies of soil and water microbial communities have also reported a “rare biosphere” which is comprised of a long tail of low abundance taxa [11], [14], [62]. The presence and function of the long tail of bacterial 16S rRNA gene OTU groups as part of the overall microbial community is hotly debated. It has been proposed that the high rate of dispersion of microbes leads to the ubiquitous presence of some taxa in nearly all environments and thus the long tail is a reflection of the majority of bacteria which do not thrive in a given environment [63], [64]. Others have proposed that the long tail is maintained due to the low predation rate on rare taxa [65]. A recent study, which measured the distribution of both OTU rDNA and rRNA in a sample, found that low abundance taxa were often more active than the highly abundant taxa, and theorized that dormancy allows some taxa to remain highly abundant under oscillating environmental conditions [66]. The ability of bacterial taxa to move along the abundance curve of through periods of dormancy and subsequent revival helps explain a number of phenomena, including seasonal succession in bacterial communities and the long tail itself [66]. This concept could potentially explain the variability in poultry litter bacterial communities seen across houses and litter conditions. Although the in-house environment is kept as stable as possible, numerous factors such feeding regimen [67] and growth of the birds themselves [68] provide stimuli for changes in the microbial composition of poultry litter in part, through the continual addition of faces.

Undoubtedly, the increased moisture in the Wet litter allows more types of bacteria to thrive. However, because moisture content also correlates with a suite of other physiochemical parameters known to play a role in microbial diversity including pH [69], [70], and availability of carbon [71], [72] and nitrogen [73] determining the predominant factor contributing to Wet litter bacterial diversity is difficult. Additionally, more types of bacterial metabolism become possible with the microenvironmental conditions provided by increased moisture content. For example, saturated or near saturated conditions can create anoxic conditions only a few centimeters from the surface, as evidenced by the occurrence of *Bacteroides* species in all the Wet libraries. Wet litter likely also exhibited a lower pH [42].

### Taxonomy of abundant 16S rRNA gene OTU Clusters

Together, the top five OTU clusters contained 29% of all sequences across the study. Clusters 1, 3, and 4 classified to the Actinobacteria, and clusters 2 and 5 to the Firmicutes. In the case of cluster 1, the RDP classifier and Seqmatch tool indicated the representative sequence belonged to the genus *Yaniella* (76% confidence) or the genus *Arthrobacter*, respectively. This inconsistency highlights the potential ambiguity associated with using short variable 16S rRNA variable regions rather than full gene sequences for classification. While OTUs clustered at 95% identity should ideally provide genus to species level resolution, this is not always the case. Further investigation of the top OTU cluster using BLAST against sequences in the nr database found that unclassified sequences, from chicken litter [47] and turkey feces [74] showed 94% identity to the representative sequence from cluster number one.

The representative sequence from the 2^nd^ most abundant OTU cluster was classified by RDP as the genus *Staphylococcus*, which was not surprising as *Staphylococcus* spp. are often found on the skin and mucous membranes of both healthy and diseased chickens [75]. BLAST analysis of this sequence found 94% homology to an uncultured Firmicute isolate from a DGGE band produced from a chicken litter sample [76]. There were also a number of BLAST hits to *Staphylococcus nepalensis* isolated from the GI tracts of monkey and pigs [4]. In the same study, Novakova *et al.* [4] found that it was impossible to differentiate between *S. xylosus* and *S. nepalensis* by biological tests alone and additional 16S rRNA gene sequencing was required to differentiate these two species. This difficulty in strain identification calls into question previous cultivation-based findings showing *S. xylosus* to be the dominant *Staphylococcus* species in poultry litter [30]. *S. xylosus* has not been identified in some of the more recent 16S rRNA gene-based studies [5], [42], but both S. xylosus and *S. nepalensis* were described in litter isolates by Nandi *et al.* [34].

The 3^rd^ largest OTU cluster was classified to the suborder Micrococcineae. Like cluster 1, unclassified poultry litter sequences showed the highest identity at 95% [47]. Also among the top hits to this cluster were sequences classified as *Brachybacterium* from sewage sludge and the Bering Sea each with 91% identity (unpub GenBank acc #: AB210986.1, GU166125.1). Near perfect correlation was seen between RDP-classified *Brachybacterium* sequences and assignment of these sequences in 16S rRNA gene OTU clusters. Interestingly, the isolates used to describe the *Brachybacterium* genus were derived from “poultry deep litter” samples taken in the 1960's [77]. In a litter study by Lu *et al.* [5], 5% of the 16S rRNA gene poultry litter clones were classified as *Brachybacterium* sp.

The representative sequence for the 4^th^ largest OTU cluster classified to the genus *Brevibacterium* with 88% confidence. Environmental sequences classified as uncultured *Brevibacterium* from poultry litter also matched with 99% identity [5]. The same study by Lu *et al.* [5] found 7% of 16S gene clones classified as *Brevibacterium sp*. In general, *Brevibacterium sp*. are not pathogenic, however there are known pathogenic species like *B. avium* [78], [79]. Comparing the representative sequence from this study to the published *B. avium* found them to be 94% similar (i.e., not the same species). The representative sequence from the 5^th^ largest cluster was not confidently classified past the family level of Bacillaceae. Both RDP seqmatch and BLAST found no high similarity hits to any classified bacteria. Like cluster 1, the most similar sequences came from previous 16S studies of poultry litter [47] (96% identity) and turkey feces [74] (92% identity). This cluster was only found in Houses 1, 2, and 3 with over 98% of the sequences contributed evenly by Houses 1 and 3.

Examining the classification of 16S rRNA gene OTU representative sequences using RDP SeqMatch and BLAST confirmed that these approaches closely matched the genus-level classifications before clustering. However, the 5^th^ largest cluster was composed of non-classifiable sequences indicating that unknown bacterial groups can be highly abundant in poultry litter. This result validates the utility of OTU-based approaches for analysis of bacterial communities. It is also encouraging that 4 of the 5 top OTUs had highly similar matches to sequences reported from previous litter studies and these bacterial taxa may comprise an important core group within poultry litter.

### Gangrenous Dermatitis connection

One goal of this study was to examine the microbial communities of poultry houses affected by recurring outbreaks of gangrenous dermatitis (GD) and compare them to communities in houses with no history of GD. Previous research has determined the putative cause(s) of GD to be associated with *Clostridium septicum*, *Clostridium perfringens* and *Staphylococcus aureus* [8], [9]. Genus level classification from the RPD classifier indicated that *Clostridium* spp. were found at low levels in both GD and non-GD houses; whereas, *Staphylococcus* was found in high abundance in all houses, and in fact represented the second largest OTU cluster. Overall, there was no clear trend in microbial community structure when comparing the frequency of 16S rRNA gene OTU clusters between in GD and non-GD houses. Because overall bacterial community structure appears to be influenced by husbandry practice, we hypothesize that recurring GD may be attributable to the existence of one or multiple low abundance taxa rather than a single high abundance taxa. This hypothesis is supported by data indicating that lower abundance taxa can represent the more active fraction of bacterial communities [66].

Within the dataset, thirteen, 16S rRNA gene OTU clusters were unique to GD houses and these comprised 1.9% of all reads (Table 4). Nearly one third of these clusters could not be assigned to the family level (≤50% confidence) and only five could be classified to the genus level. The majority of the GD unique clusters were derived from sequences in Wet libraries. This is not surprising considering the higher bacterial diversity of in wet litter (Fig. 1 B&D, Fig. 4) and highlights the potential for wet litter environments to harbor pathogens. Examination of the 16S rRNA gene OTU clusters with high confidence genus-level RDP classifications shows that *Anaerococcus* spp. were the first and tenth largest unique GD clusters (the 30^th^ and the 152^nd^ most abundant clusters overall, respectively). *Anaerococcu*s spp. belong to a larger loosely defined group of Gram-positive anerobic cocci (GPAC) which make up a large part of human microbial flora [80], [81]. Many *Anaerococcus* strains have clinical significance having been isolated from the penis and vagina microbiomes [18], [82] and numerous diabetic ulcers and other infections [83]. Another GD unique OTU was classified as *Enterococcus*. Although sequences classifying to the *Enterococcus* were present in all samples, this particular 16S rRNA gene OTU cluster was found predominantly in the GD houses. Only one sequence from each non-GD house recruited to this cluster so the cluster was considered unique, and likely represents a different species or strain than the ones found in the non-GD houses. As a genus, *Enterococcus* has gained attention in recent years due to the isolation of increasingly antibiotic resistant stains from both clinical and industrial settings [84], [85]. *Enterococcus* species *faecium* and *faecalis* with resistance to numerous antibiotics have been isolated from both poultry litter and poultry transport containers [85], [86].

**Table 4:** 16S rRNA gene OTUs unique to houses with history of gangrenous dermatitis.

			Ribosomal Database Classification of representative OTU sequence (% confidence)				
Cluster number^a^	# of seqs	Majority Dry or Wet derived	Phylum	Class	Order	Family	Genus
30	129	W	*Firmicutes* (100)	*Clostridia* (100)	*Clostridiales* (100)	*Incertae Sedis XI* (100)	*Anaerococcus* (100)
69	48	D	*Actinobacteria* (100)	*Actinobacteria* (100)	*Actinomycetales* (100)	Pseudonocardineae (85)	*Saccharomonospora* (35)
69	48	W	*Firmicutes* (95)	*Bacilli* (83)	*Bacillales* (74)	*Bacillaceae* (46)	*Halalkalibacillus* (13)
91	31	W	*Bacteroidetes* (80)	*Sphingobacteria* (42)	*Sphingobacteriales* (42)	*Saprospiraceae* (36)	*Haliscomenobacter* (22)
104	26	W	*Deinococcus-Thermus* (26)	*Deinococci* (26)	*Thermales* (11)	*Thermaceae* (11)	*Vulcanithermus* (8)
107	24	W	*Firmicutes* (97)	*Bacilli* (95)	*Bacillales* (92)	*Bacillaceae* (80)	*Salirhabdus* (8)
114	23	W	*Bacteroidetes* (100)	*Bacteroidia* (78)	*Bacteroidales* (78)	*Bacteroidaceae* (75)	*Bacteroides* (75)
114	23	W	*Firmicutes* (100)	*Bacilli* (100)	*Lactobacillales* (100)	*Enterococcaceae* (100)	*Enterococcus* (100)
140	17	W	*Bacteroidetes* (93)	*Bacteroidia* (77)	*Bacteroidales* (77)	*Porphyromonadaceae* (77)	*Dysgonomonas* (45)
152	15	W	*Firmicutes* (96)	*Clostridia* (96)	*Clostridiales* (96)	*Incertae Sedis XI* (96)	*Anaerococcus* (95)
161	14	W	*Firmicutes* (100)	*Clostridia* (100)	*Clostridiales* (100)	*Incertae Sedis XI* (91)	*Tepidimicrobium* (45)
170	13	W	*Proteobacteria* (99)	*Alphaproteobacteria* (90)	*Rhodospirillales* (75)	*Rhodospirillaceae* (75)	*Fodinicurvata* (62)
170	13	D	*Actinobacteria* (93)	*Actinobacteria* (93)	*Actinomycetales* (90)	*Microbacteriaceae* (40)	*Okibacterium* (33)

a ) ranked by abundance.

This cross sectional study of poultry litter within a range of house environments provides a starting point for further investigations into the influence of litter microbial communities on poultry health. In the particular case of gangrenous dermatitis, longitudinal sampling over a GD season could potentially capture the shifts in the microbial community leading up to a GD outbreak and the subsequent return to ‘normal’ non-disease conditions. In addition to temporal sampling, increasing the sample size to include a wider variety of housing conditions will help further our understanding of how the poultry house environment influences the litter microbial community. Taking samples from several houses on a particular farm (assuming a similar construction and housing set-up) could reveal how much variation exists between litter samples collected from houses in close proximity to one another. Finally, microbiome analyses of poultry feces and chicken body sites may also to help to elucidate the etiology of GD and other poultry diseases.

## Supporting Information

Table S1 16S rRNA gene primer sequences used with poultry litter samples.
(PDF)

Table S2 Representative Sequences for the Top 50 OTU Clusters.
(XLS)

Table S3 Assignment details for the Representative Sequences of the Top 50 Clusters. Classifier: RDP Naive Bayesian rRNA Classifier Version 2.2, March 2010. Taxonomical Hierarchy: RDP training set 6, based on nomenclatural taxonomy and Bergey's Manual. Confidence threshold: 50%.
(XLS)

## References

[1] Ollinger M, Macdonald JM, Madison M (2005). Technological change and economies of scale in U.S. poultry processing.. The American Journal of Agricultural Economics.

[2] United States Department of Agriculture NSS (2010). Poultry-production value 2009 summary.. United States Department of Agriculture, National Statistics Service.

[3] Boyd W (2001). Making Meat: Science, technology, and American poultry production.. Technology and Culture.

[4] Novakova D, Pantucek R, Petras P, Koukalova D, Sedlacek I (2006). Occurance of *Staphylococcus nepalensis* strains in different sources including human clinical material.. FEMS Microbiol Lett.

[5] Lu J, Sanchez S, Hofacre C, Maurer JJ, Harmon BG (2003). Evaluation of Broiler Litter with Reference to the Microbial Composition as Assessed by Using 16S rRNA and Functional Gene Markers.. Applied and Environmental Microbiology.

[6] Collins SP, Lang M (2006).

[7] Frazier MN, Parizek WJ, Garner E (1964). Gangrenous dermatitis in chickens.. Avian Diseases.

[8] Wilder TD, Barbaree JM, Macklin KS, Norton RA (2001). Differences in the pathogenicity of various bacterial isolates used in an induction model for gangrenous dermatitis in broiler chickens.. Avian Diseases.

[9] Willoughby DH, Bickford AA, Cooper GL, Charlton BR (1996). Periodic recurrence of gangrenous dermatitis associated with Clostridium septicum in a broiler chicken operation.. Journal of veterinary diagnostic investigation : official publication of the American Association of Veterinary Laboratory Diagnosticians, Inc.

[10] Kaul M, Tanwani SK, Sharda R (2001). Preliminary studies on bacterin against gangrenous dermatitis.. Indian Veterinary.

[11] Galand PE, Casamayor EO, Kirchman DL, Lovejoy C (2009). Ecology of the rare microbial biosphere of the Arctic Ocean.. Proceedings of the National Academy of Sciences of the United States of America.

[12] Hollister EB, Engledow AS, Hammett AJ, Provin TL, Wilkinson HH (2010). Shifts in microbial community structure along an ecological gradient of hypersaline soils and sediments.. The ISME journal.

[13] Rusch DB, Halpern AL, Sutton G, Heidelberg KB, Williamson S (2007). The Sorcerer II Global Ocean Sampling expedition: northwest Atlantic through eastern tropical Pacific.. PLoS biology.

[14] Sogin ML, Morrison HG, Huber JA, Mark Welch D, Huse SM (2006). Microbial diversity in the deep sea and the underexplored “rare biosphere”.. Proceedings of the National Academy of Sciences of the United States of America.

[15] Teixeira LC, Peixoto RS, Cury JC, Sul WJ, Pellizari VH (2010). Bacterial diversity in rhizosphere soil from Antarctic vascular plants of Admiralty Bay, maritime Antarctica.. The ISME journal.

[16] Costello EK, Lauber CL, Hamady M, Fierer N, Gordon JI (2009). Bacterial community variation in human body habitats across space and time.. Science (New York, NY).

[17] Eckburg PB, Bik EM, Bernstein CN, Purdom E, Dethlefsen L (2005). Diversity of the human intestinal microbial flora.. Science (New York, NY).

[18] Price LB, Liu CM, Johnson KE, Aziz M, Lau MK (2010). The effects of circumcision on the penis microbiome.. PloS one.

[19] Ravel J, Gajer P, Abdo Z, Schneider GM, Koenig SS (2010). Microbes and Health Sackler Colloquium: Vaginal microbiome of reproductive-age women.. Proceedings of the National Academy of Sciences of the United States of America.

[20] van Elsas JD, Smalla K, Hurst CJ, Knudsen GR, McInerney MJ, bach LDS-, Walter MV (1997). Methods for sampling soil microbes.. Manual of environmental micobiology.

[21] Williamson KE, Wommack KE, Radosevich M (2003). Sampling Natural Viral Communities from Soil for Culture-Independent Analyses.. Applied and Environmental Microbiology.

[22] Hamady M, Walker JJ, Harris JK, Gold NJ, Knight R (2008). Error-correcting barcoded primers for pyrosequencing hundreds of samples in multiplex.. Nature Methods.

[23] Schloss PD, Handelsman J (2005). Introducing DOTUR, a Computer Program for Defining Operational Taxonomic Units and Estimating Species Richness.. Applied and Environmental Microbiology.

[24] Wang Q, Garrity GM, Tiedje JM, Cole JR (2007). Naive Bayesian classifier for rapid assignment of rRNA sequences into the new bacterial taxonomy.. Applied and Environmental Microbiology.

[25] Edgar RC, Haas BJ, Clemente JC, Quince C, Knight R (2011). UCHIME improves sensitivity and speed of chimera detection.. Bioinformatics.

[26] Pavoine S, Dufour AB, Chesses D (2004). From dissimilarities among species to dissimilarities among communities: a double principle coordinate analysis.. Journal.

[27] Team RDC (2010). R: a language and environment for statistical computing..

[28] Dray S, Dufour AB (2007). Journal of Statistical Software.

[29] Fries R, Akcan M, Bandick N, Kobe A (2005). Microflora of two different types of poultry litter.. British poultry science.

[30] Martin SA, McCann MA (1998). Microbiological study of Georgia poultry litter.. Journal of Applied Poultry Research.

[31] Omeira N, Barbour EK, Nehme PA, Hamadeh SK, Zurayk R (2006). Microbiological and chemical properties of litter from different chicken types and production systems.. The Science of the total environment.

[32] Terzich M, Pope MJ, Cherry TE, Hollinger J (2000). Survey of pathogens in poultry litter in the United States.. Journal of Applied Poultry Research.

[33] Thaxton YV, Balzli CL, Tankson JD (2003). Relationship of Broiler Flock Numbers to Litter Microflora.. Journal of Applied Poultry Research.

[34] Nandi S, Maurer JJ, Hofacre C, Summers AO (2004). Gram-positive bacteria are a major reservoir of Class 1 antibiotic resistance integrons in poultry litter.. Proceedings of the National Academy of Sciences of the United States of America.

[35] Kabell S, Handberg K, Li Y, Kusk M, Bisgaard M (2005). Detection of vvIBDV in Vaccinated SPF Chickens.. Acta Veterinaria Scandinavica.

[36] Khurana S, Suguitan AL, Rivera Y, Simmons CP, Lanzavecchia A (2009). Antigenic Fingerprinting of H5N1 Avian Influenza Using Convalescent Sera and Monoclonal Antibodies Reveals Potential Vaccine and Diagnostic Targets.. PLoS Medicine.

[37] Williamson KE, Radosevich M, Smith DW, Wommack KE (2007). Incidence of lysogeny within temperate and extreme soil environments.. Environmental microbiology.

[38] Williamson KE, Radosevich M, Wommack KE (2005). Abundance and Diversity of Viruses in Six Delaware Soils.. Applied and Environmental Microbiology.

[39] Wommack KE, Colwell RR (2000). Virioplankton: Viruses in aquatic ecosystems.. Microbiol Molec Biol Rev.

[40] Helton RR, Liu L, Wommack KE (2006). Assessment of Factors Influencing Direct Enumeration of Viruses within Estuarine Sediments.. Applied and Environmental Microbiology.

[41] Hewson I, Fuhrman JA (2003). Viriobenthos production and virioplankton sorptive scavenging by suspended sediment particles in coastal and pelagic waters.. Microbial ecology.

[42] Lovanh N, Cook KL, Rothrock MJ, Miles DM, Sistani K (2007). Spatial shifts in microbial population structure within poultry litter associated with physicochemical properties.. Poultry science.

[43] Margulies M, Egholm M, Altman WE, Attiya S, Bader JS (2005). Genome Sequencing in Open Microfabricated High Density Picoliter Reactors.. Nature.

[44] Cole JR, Wang Q, Cardenas E, Fish J, Chai B (2009). The Ribosomal Database Project: improved alignments and new tools for rRNA analysis.. Nucleic acids research.

[45] Larsen N, Olsen GJ, Maidak BL, McCaughey MJ, Overbeek R (1993). The ribosomal database project.. Nucleic acids research.

[46] Claesson MJ, O'Sullivan O, Wang Q, Nikkilä J, Marchesi JR (2009). Comparative Analysis of Pyrosequencing and a Phylogenetic Microarray for Exploring Microbial Community Structures in the Human Distal Intestine.. PLoS One.

[47] Enticknap JJ, Nonogaki H, Place AR, Hill RT (2006). Microbial Diversity Associated with Odor Modification for Production of Fertilizers from Chicken Litter.. Applied and Environmental Microbiology.

[48] Frias-Lopez J, Shi Y, Tyson GW, Coleman ML, Schuster SC (2008). Microbial community gene expression in ocean surface waters.. Proceedings of the National Academy of Sciences of the United States of America.

[49] Schloss PD, Handelsman J (2006). Toward a Census of Bacteria in Soil.. PLoS Computational Biology.

[50] Vandamme P, De Ley J (1991). Proposal for a New Family, Campylobacteraceae.. International Journal of Systemic Bacteriology.

[51] Vandenberg O, Dediste A, Houf K, Ibekwem S, Souayah H (2004). Arcobacter species in humans.. Emerging infectious diseases.

[52] Wybo I, Breynaert J, Lauwers S, Lindenburg F, Houf K (2004). Isolation of Arcobacter skirrowii from a Patient with Chronic Diarrhea.. Journal of clinical microbiology.

[53] Gude A, Hillman TJ, Helps CR, Allen VM, Corry JE (2005). Ecology of Arcobacter species in chicken rearing and processing.. Letters in applied microbiology.

[54] Van Driessche E, Houf K (2007). Discrepancy between the occurrence of Arcobacter in chickens and broiler carcass contamination.. Poultry science.

[55] Ho HT, Lipman LJ, Gaastra W (2008). The introduction of Arcobacter spp. in poultry slaughterhouses.. International journal of food microbiology.

[56] Bae HS, Rash BA, Rainey FA, Nobre MF, Tiago I (2007). Description of Azospira restricta sp. nov., a nitrogen-fixing bacterium isolated from groundwater.. International Journal of Systematic and Evolutionary Microbiology.

[57] Coates JD, Michaelidou U, Bruce RA, O'Connor SM, Crespi JN (1999). Ubiquity and diversity of dissimilatory (per)chlorate-reducing bacteria.. Applied and Environmental Microbiology.

[58] Tan Z, Reinhold-Hurek B (2003). Dechlorosoma suillum Achenbach et al. 2001 is a later subjective synonym of Azospira oryzae Reinhold-Hurek and Hurek 2000.. International Journal of Systematic and Evolutionary Microbiology.

[59] Hunter WJ (2007). An Azospira oryzae (syn Dechlorosoma suillum) strain that reduces selenate and selenite to elemental red selenium.. Current microbiology.

[60] Van Trump JI, Coates JD (2009). Thermodynamic targeting of microbial perchlorate reduction by selective electron donors.. The ISME journal.

[61] McGill BJ, Etienne RS, Gray JS, Alonso D, Anderson MJ (2007). Species abundance distributions: moving beyond single prediction theories to integration within an ecological framework.. Ecology Letters.

[62] Elshahed MS, Youssef NH, Spain AM, Sheik C, Najar FZ (2008). Novelty and uniqueness patterns of rare members of the soil biosphere.. Applied and Environmental Microbiology.

[63] Finlay BJ (2002). Global dispersal of free-living microbial eukaryote species.. Science (New York, NY).

[64] Finlay BJ, Clarke KJ (1999). Ubiquitous dispersal of microbial species.. Nature.

[65] Pedros-Alio C (2006). Marine microbial diversity: can it be determined?. Trends in microbiology.

[66] Jones SE, Lennon JT (2010). Dormancy contributes to the maintenance of microbial diversity.. Proceedings of the National Academy of Sciences of the United States of America.

[67] Johansen CH, Bjerrum L, Pedersen K (2007). Impact of salinomycin on the intestinal microflora of broiler chickens.. Acta Vet Scand.

[68] Lu J, Idris U, Harmon B, Hofacre C, Maurer JJ (2003). Diversity and succession of the intestinal bacterial community of the maturing broiler chicken.. Applied and Environmental Microbiology.

[69] Hogberg MN, Hogberg P, Myrold DD (2007). Is microbial community composition in boreal forest soils determined by pH, C-to-N ratio, the trees, or all three?. Oecologia.

[70] Lauber CL, Hamady M, Knight R, Fierer N (2009). Pyrosequencing-Based Assessment of Soil pH as a Predictor of Soil Bacterial Community Structure at the Continental Scale.. Applied and Environmental Microbiology.

[71] Monard C, Binet F, Vandenkoornhuyse P (2008). Short-term response of soil bacteria to carbon enrichment in different soil microsites.. Applied and Environmental Microbiology.

[72] Wawrik B, Kerkhof L, Kukor J, Zylstra G (2005). Effect of Different Carbon Sources on Community Composition of Bacterial Enrichments from Soil.. Applied and Environmental Microbiology.

[73] Mendum TA, Sockett RE, Hirsch PR (1999). Use of molecular and isotopic techniques to monitor the response of autotrophic ammonia-oxidizing populations of the beta subdivision of the class proteobacteria in arable soils to nitrogen fertilizer.. Applied and Environmental Microbiology.

[74] Lu J, Domingo JS (2008). Turkey fecal microbial community structure and functional gene diversity revealed by 16S rRNA gene and metagenomic sequences.. Journal of microbiology (Seoul, Korea).

[75] Smyth DS, Feil EJ, Meaney WJ, Hartigan PJ, Tollersrud T (2009). Molecular genetic typing reveals further insights into the diversity of animal-associated Staphylococcus aureus.. Journal of medical microbiology.

[76] Rothrock MJ, Cook KL, Warren JG, Sistani K (2008). The effect of alum addition on microbial communities in poultry litter.. Poultry science.

[77] Collins L, Sumpter D (2007). The feeding dynamics of broiler chickens.. Journal of the Royal Society Interface.

[78] Onraedt A, Soetaert W, Vandamme E (2005). Industrial importance of the genus Brevibacterium.. Biotechnology Letters.

[79] Pascual C, Collins MD (1999). Brevibacterium avium sp. nov., isolated from poultry.. International Journal of Systematic Bacteriology.

[80] Murdoch DA (1998). Gram-positive anaerobic cocci.. Clinical microbiology reviews.

[81] Song Y, Liu C, McTeague M, Finegold SM (2003). 16S ribosomal DNA sequence-based analysis of clinically significant gram-positive anaerobic cocci.. Journal of clinical microbiology.

[82] Srinivasan S, Fredricks DN (2008). The Human Vaginal Bacterial Biota and Bacterial Vaginosis.. Interdisciplinary Perspectives on Infectious Diseases 2008.

[83] Song Y, Liu C, Finegold SM (2007). Peptoniphilus gorbachii sp. nov., Peptoniphilus olsenii sp. nov., and Anaerococcus murdochii sp. nov. isolated from clinical specimens of human origin.. Journal of clinical microbiology.

[84] Ghidan A, Kaszanyitzky EJ, Dobay O, Nagy K, Amyes SG (2008). Distribution and genetic relatedness of vancomycin-resistant enterococci (VRE) isolated from healthy slaughtered chickens in Hungary from 2001 to 2004.. Acta Veterinaria Hungarica.

[85] Hayes JR, English LL, Carr LE, Wagner DD, Joseph SW (2004). Multiple-Antibiotic Resistance of Enterococcus spp. Isolated from Commercial Poultry Production Environments.. Applied and Environmental Microbiology.

[86] Graham JP, Evans SL, Price LB, Silbergeld EK (2009). Fate of antimicrobial-resistant enterococci and staphylococci and resistance determinants in stored poultry litter.. Environmental research.

