# Supplementary material for: Impacts of Poultry House Environment on Poultry Litter Bacterial Community Composition
Source: PLoS One. 2011 Sep 16;6(9):e24785. doi: 10.1371/journal.pone.0024785 (PMC3174962; doi:10.1371/journal.pone.0024785)
Supplement: Table S1 — 16S rRNA gene primer sequences used with poultry litter samples. (PDF) [file pone.0024785.s001.pdf]

Table S1. 16S rRNA gene primer sequences used with poultry litter samples.

Sample name		Primer Sequence (5^1-3^1)[a]
Dry 1	338R	GCCTCCCTCGCGCCATCAGACTCGTCTCATGCTGCCTCCCGTAGGAGT
Dry 2		GCCTCCCTCGCGCCATCAGACTGACTGCATGCTGCCTCCCGTAGGAGT
Dry 3		GCCTCCCTCGCGCCATCAGACTCGTCTCATGCTGCCTCCCGTAGGAGT
Dry 4		GCCTCCCTCGCGCCATCAGACTGCTCTCATGCTGCCTCCCGTAGGAGT
Wet 1		GCCTCCCTCGCGCCATCAGACTGACTGCATGCTGCCTCCCGTAGGAGT
Wet 2		GCCTCCCTCGCGCCATCAGAGAGACTGCATGCTGCCTCCCGTAGGAGT
Wet 3		GCCTCCCTCGCGCCATCAGAGACGTCTCATGCTGCCTCCCGTAGGAGT
Wet 4		GCCTCCCTCGCGCCATCAGAGAGCTCTCATGCTGCCTCCCGTAGGAGT
All samples	27F	GCCTTGCCAGCCCGCTCAGTCAGAGTTTGATCCTGGCTCAG

[a] blue region is the 454 linker, red region is the barcode, green region is the primer sequence
